# Therapy for Children with Henoch-Schonlein Purpura Nephritis: A Systematic Review

**DOI:** 10.1100/tsw.2007.23

**Published:** 2007-01-10

**Authors:** Marco Zaffanello, Milena Brugnara, Massimo Franchini

**Affiliations:** ^1^ Department of Paediatrics, University of Verona, Verona, Italy; ^2^ Transfusion Service, Verona Hospital, Verona, Italy

**Keywords:** Henoch-Schonlein purpura, nephritis, therapy, children

## Abstract

Although severe kidney involvement in children with Henoch-Shonlein purpura (HSP) is rarer than that in adults, morbidity should not be underevaluated and follow-up is mandatory. Some drugs are introduced as well-defined treatment options, others can be promising therapeutic alternatives.

Therapy of HSP nephritis in children can range from simply steroids to combined immunosuppressant treatments. The prophylactic treatment for renal complication of patients with HSP has been sometimes suggested, but with conflicting results and ultimately not clearly proven. The treatment of overt HSP nephritis includes steroids and other immunosuppressant drugs. Methylprednisolone pulse therapy and prednisone per os are tested drugs. These steroids could be used in combination with other immunosuppressant drugs, such as cyclosporin A and cyclophosphamide. Unfortunately, of these two drugs, only cyclophosphamide is demonstrated as effective in a recent randomized controlled trial. However, since there are insufficient data and unstructured study designs, ACE-I, azathioprine, mycophenolate mofetil, and urokinase need to be more tested in childhood HSP nephritis. In addition to drugs, other techniques are used to treat the severe form of nephritis. Of these, in a multicenter study, plasmapheresis demonstrated efficacy in delaying the progression of kidney disease. However, no convincing studies have been made to date concerning either intravenous immunoglobulin, factor XIII administration, antioxidant vitamin E, and fish oil to treat HSP nephritis.

## INTRODUCTION

Henoch-Schonlein purpura (HSP) is an immunologically mediated disease associating purpura plus urticaria, characteristically affecting lower limbs, buttocks, and elbows. Frequently, HSP leads to abdominal pain and intestinal bleeding, renal involvement and joint pain. The IgA deposits in vessel walls, associated with the characteristic signs of small-vessel vasculitis, are the *sine qua non* in the diagnosis[1]. Although the reported rate of renal changes is 17% of HSP patients, less than 2% can develop a serious kidney complication associated with long-term morbidity[2]. Gastrointestinal involvement, if present, may require a surgical or laparotomy approach. In addition, pulmonary hemorrhage is extremely rare[3,4], as well as chylothorax[5] and bilateral ureteral obstruction from ureteral ischemia[6]. Routine laboratory test results are usually within reference ranges. Although the diagnosis is clinical and not based on laboratory evaluation, additional laboratory tests that can be helpful in narrowing the differential diagnosis include the following: assessment of antistreptolysin-O (ASO) titer, Monospot test, antinuclear antibody (ANA) test, Rheumatoid Factor (RF) test, determination of C3/C4 levels, measurement of the Prothrombin Time (PT), measurement of the activated Partial Thromboplastin Time (aPTT), and blood cultures. Finally, laboratory findings of low $C_3$ levels, leucopenia, and thrombocytopenia are rarely described in this disease[7].

HSP is primarily a rheumatic disease of children. Renal involvement is a common finding in children with HSP[8], although most of them recover completely. Nevertheless, the risk of progression to end-stage renal disease may exist. The main clinical signs of rapidly progressive HSP nephritis are edema, hypertension, gross hematuria, and oliguria[9]. Renal involvement in HSP can be also quantified by means of a kidney biopsy, demonstrating positive mesangial staining and positive anti-IgA antisera on immunofluorescence[10]. Furthermore, a persistent or increased fibrinogen deposition in the kidney during the course of the disease is closely associated with severe renal injury[11]. Finally, the majority of patients develop a chronic glomerular IgA nephropathy many years after the initial vasculitis episode[12].

The HSP disease is quite rare. For example, the yearly incidence of nephrotic-range HSP in Finland was 2 per million children under 15 years old[13]. The annual incidence of HSP nephritis in Italian children was 1.9 cases per million[14]. Most patients with HSP (33.5%) developed renal involvement 3 days to 17 months after the onset of the disease[15]. In another study, 49% of HSP patients developed renal involvement, but almost all (97%) within 3 months of disease onset[16]. In particular, 20.8% of HSP in childhood ranged from the more common microhematuria to the less common nephrotic syndrome (NS)[17]. Long-term renal impairment was lower (1.6%) in subjects who had only abnormal urinary findings at examination and higher (19.5%) in those who developed nephritis or NS. A rare, serious, and later complication was end-stage kidney disease[18].

In adults suffering from HSP, the outcome was relatively poor. Regarding renal function, 11% reached end-stage renal failure, 13% exhibited severe renal failure (CrCl < 30 ml/min), and 14% showed moderate renal insufficiency (CrCl < 50 ml/min)[19]. In severe cases, the institution of early and aggressive immunosuppressive treatment improves long-term kidney survival[8]. Contrary to that seen in adults, the outcome in HSP children showed better. In fact, the overall percentage of children who developed long-term renal impairment was only 1.8%[20].

Although severe kidney involvement in children with HSP is rarer than that in adults, the morbidity should not be underevaluated and follow-up may be mandatory. Some drugs are introduced as well-defined treatment options, others can be promising therapeutic alternatives. The present review summarizes the drugs and the new therapeutic strategies in childhood HSP nephritis.

## TREATMENTS

Table 1 summarizes the most interesting pharmacological therapeutic strategies obtained from the literature.

### Steroids

For many years, steroids have been used as a first-line treatment for several nephropathies in children. The proposed treatment of HSP nephritis with steroids could be prophylactic or to treat developed nephropathy. As prophylactic measures against nephropathy, the treatment of HSP patients with steroids has shown conflicting results. In a retrospective study, patients with HSP, without clinical signs of nephropathy, were

**TABLE 1**
**Treatment of Childhood HSP Nephritis: Results of the Literature**

| Study ID | Study Design | Treatment | Control | Subjects (n°) | Duration Treatment | Duration Follow-up | Objective | Evidence |
|---|---|---|---|---|---|---|---|---|
| Buchanec, 1988 | Retrospective | Prednisone | Yes | 33 | 21 days | — | Effectiveness in prevention of nephropathy | Needs more studies |
| Saulsbury, 1993 | Retrospective | Prednisone | Yes | 50 | — | — | Effectiveness in prevention of nephropathy | No |
| Reinehr, 2000 | Retrospective | Prednisone | Yes | 101 | 1 week | 1.5 years | Effectiveness in prevention of nephropathy | Yes |
| Huber, 2004 | Randomized controlled trial | Prednisone | Placebo | 40 | 2 weeks | 1 year | Early treatment and reduction of renal complication | None at 1 year |
| Ronkainen, 2006 | Randomized double-blind, placebo-controlled trial | Prednisone | Placebo | 171 | 4 weeks | 6 months | Efficacy in preventing and treating renal symptoms | Effective in altering (but not preventing) |
| Niaudet, 1998 | Uncontrolled, prospective study | Methylprednisolone (and prednisone) | No | 38 | 3.5 months | 1–16 years | Effectiveness in at risk nephropathy | Effective in patients with progression |
| Ronkainen, 2003 | Clinical trial | Cyclosporin A (and ACE-I) | No | 7 | 1.7–2.1 years | 6 years | Efficacy in nephritic form | Effective |
| Someya, 2004 | Case report | Cyclosporin A | No | 1 | 6 months | 1 year | Effectiveness in severe nephritis steroid resistant | Effective, side effect CyA-dependent nephritis |
| Shin, 2005 | Retrospective | Cyclosporin A (and steroid) | No | 7 | 8–12 months | 5.5 ± 2.5 years | Efficacy to induce remission in nephropathy | May be beneficial |
| Shin, 2005 | Retrospective | Cyclosporin A (and steroid) | No | 8 | - | 3.8 years | Therapeutic role in nephritis | May regress renal pathology |
| Tarshish, 2004 | Randomized controlled trial | Cyclophosphamide | Supportive therapy | 56 | 42 days | 5 years | Efficacy in severe nephritis | None at 4–11 years |
| Flynn, 2001 | Clinical trial, retrospective | Cyclophosphamide (and methylprednisolone or prednisone) | No | 12 | 12 weeks | — | Revision of a treatment protocol | Safe and effective |
| Tanaka, 2003 | Clinical trial | Cyclophosphamide and prednisolone | No | 9 | 8 weeks | 78 months | Efficacy in severe proteinuric nephritis | May be beneficial |
| Kawasaki, 2004 | Clinical trial | Cyclophosphamide (and methylprednisolone and urokinase) | Methylprednisolone and urokinase | 37 | 12 weeks | 24 months | Effectiveness in severe nephritis | Effective |
| Oner, 1995 | Clinical trial | Cyclophosphamide (and dipyridamole and methylprednisolone and prednisolone) | No | 12 | 6 months | 9–39 months | Effectiveness | May be effective |

**TABLE 1 continued**

| Study ID | Study Design | Treatment | Control | Subjects (n°) | Duration Treatment | Duration Follow-up | Objective | Evidence |
|---|---|---|---|---|---|---|---|---|
| Iijima, 1998 | Retrospective | Cyclophosphamide (and prednisone and dipyridamole and heparin/warfarin) | No | 14 | 8 weeks | 7.5 ± 0.9 years | Effectiveness in histologically severe nephritis | Could be effective |
| Dixit, 2004 | Case reports | ACE-I (and fish oil) | No | 5 | 21–83 weeks | 21–83 weeks | Progression of nephropathy | Containment of disease |
| Bergstein, 1998 | Clinical trial | Azathioprine (and methylprednisolone and prednisone) | Azathioprine (and prednisone) | 21 | 15 months | 32 months | Effectiveness in severe nephritis | Effective |
| Singh, 2002 | Retrospective | Azathioprine (and methylprednisolone or dexamethasone andprednisolone) | No | 9 | 6–24 months | 6 months to 1.5 years | Analyses of clinical course | Improve the outcome in severe nephritis at 5 years |
| Foster, 2000 | Retrospective | Azathioprine (and prednisone) | No | 20 | — | — | Prevention of chronic changes | Early treatment |
| Shin, 2005 | Retrospective | Azathioprine (mycophenolate mofetil and steroid) | Yes (steroid alone) | 20 | 8 months | 1–14 years | Effectiveness in severe nephritis | May be beneficial |
| Filler, 2003 | One case into a clinical trial | Mycophenolate mofetil | No | 1 | — | 138 days | Effectiveness in severe atypical nephritis | Safe and effective |
| Kawasaki, 2003 | Uncontrolled, retrospective | Methylprednisolone (and urokinase and prednisolone) | No | 56 | 3 day and (7 days and 6 months) | 9.7 ± 6.0 years | Effectiveness in severe form of nephritis | May be beneficial |
| Kawasaki, 2004 | Clinical trial | Cyclophosphamide (and methylprednisolone and urokinase) | Methylprednisolone and urokinase | 37 | 12 weeks and 3 days and 7 days | 24 months | Effectiveness in severe nephritis | Effective |

treated with prednisone for an average of 21 days. Compared with untreated patients, they showed five times less incidence of nephropathy[21]. Another retrospective study confirmed this finding. In fact, children with HSP and serious abdominal pain treated early with prednisone (2–5 mg/kg/day) for 1 week showed a lower risk of renal involvement than controls[22]. On the other hand, retrospective evaluation of an early steroid treatment administered during the acute phase of HSP in 20 children did not prevent delayed nephritis compared with 30 children who were not treated[23]. In another study, a randomized, placebo-controlled trial investigated whether early steroid administration reduced the rate of complications in HSP children. The prednisone therapy (2 mg/kg/day) for 1 week, with prednisone weaning over a second week, did not appear to reduce the risk of renal involvement at follow-up after 1 year[24]. Unfortunately, most of these studies used only a short steroid treatment limited to 1–2 weeks. We speculate that the steroid prophylaxis performed could be insufficient to obtain an adequate immunosuppression. Only one older study[21] was performed for a longer time period and obtained results successfully. However, a randomized controlled trial[24] showed that early short treatment did not prevent further renal complication. Finally, a new randomized controlled trial was performed in 171 children with severe HSP. The results of this study showed that an early prednisone therapy should be effective in altering, but not preventing, the course of renal involvement of the disease[25].

Steroid treatment performed with high intravenous doses (pulses) and/or long-term oral administration are the treatment of choice in acute HSP glomerulonephritis (GN) in children. Methylprednisolone pulse was an effective therapy in the case of severe GN. This is suggested from many studies that report on steroid pulses alone or combined with oral therapy. Furthermore, patients with severe NS and/or crescentic glomeruli were treated with intravenous pulse methylprednisolone at a dose of 1,000 mg/1.73 mq intravenously (three doses at alternate days) followed by oral prednisone for 3.5 months[26]. High intravenous doses have been shown to be effective, particularly on patients with severe nephritic HSP episodes.

Accordingly, the therapy with steroids can be used alone or combined/replaced with other drug categories on the basis of the disease's response (i.e., cyclosporin, cyclophosphamide, azathioprine, or mycophenolate mofetil).

## Cyclosporin A and Steroids

Cyclosporin A (CyA) is an effective immunosuppressive drug used for different immunomediated glomerular diseases. In particular, CyA therapy is frequently considered in the case of severe steroid-resistant HSP GN. Several investigations in children have shown its valuable results. In a clinical trial, young patients (7.2–15.2 years old) showed stable remission after a mean follow-up of 6.0 years. The initial CyA dose ranged from 4–8 mg/kg/day (blood level kept at 150–200 μg/l). During the maintenance phase, the dose of CyA was 1–5 mg/kg/day (blood level kept at 80–100 μg/l). These patients responded to the CyA treatment within a mean time of 1.4 months. However, some patients seemed to develop CyA-dependent nephritis[27,28]. In two retrospective investigations by the same authors, CyA at a starting dose of 5 mg/kg/day and steroid therapy for 8–12 months should be effective to ameliorate the renal symptoms of HSP patients with nephrotic-range proteinuria[29,30]. We can conclude that, unfortunately, the clinical experience with CyA in HSP nephritis is poor and limited to outside a clinical trial.

## Cyclophosphamide and Steroids

Cyclophosphamide belongs to a class of immunosuppressant drugs known as alkylating agents. Cyclophosphamide has been widely used as oral or intravenous formulations to treat nephritic/nephrotic disorders. This drug can be used alone or in combination with others.

The effectiveness of this drug is offered from several reports. In a recent randomized, controlled trial of cyclophosphamide therapy, pediatric patients with severe HSP nephritis received oral cyclophosphamide (90 mg/mq/day) for 42 days. The status at final follow-up was largely of a full recovery (48.2%), but the remaining patients showed persistent abnormalities (39.3%) and end-stage renal disease (12.5%). The conclusion of this study was in agreement with a failure of the therapy to obtain a complete recovery of the patients[31].

Cyclophosphamide should be also used in association with steroids. This combination resulted in an improvement of renal function and remission of NS during systemic vasculitis[32]. In a retrospective clinical trial, the treatment of HSP GN with oral cyclophosphamide (2 mg/kg/day, 12 weeks) and pulse methylprednisolone (or oral prednisone) significantly reduced the severe proteinuria[33]. In addition, in a clinical trial, a combined 8-week course of cyclophosphamide (2 mg/kg/day) with oral prednisolone (1.5 mg/kg/day) in children with severe HSP nephritis was also of beneficial effect[34].

Cyclophosphamide may also be used associated with multiple drug therapy. Triple therapy with oral cyclophosphamide (2.5 mg/kg/day, 12 weeks) along with methylprednisolone (30 mg/kg/day, maximum 1 g, 3 consecutive days) and, finally, intravenous pulse therapy of urokinase (5,000 U/kg/day, maximum 180,000 U, 7 consecutive days) was effective for patients with severe HSP nephritis. As shown by this clinical trial, after 6 months of treatment, the children showed a reduction of both mean urinary protein excretion and mesangial IgA deposition[35]. In another clinical trial, a fourth therapy with cyclophosphamide (2 months duration) combined with intravenous pulse methylprednisolone (3 days), oral prednisolone (3 months), and oral dipyridamole (6 months) allowed a normalization of the glomerular filtration rate (GFR) in almost all HSP patients aged 6–14 years. At follow-up, only a few patients showed persistent proteinuria, hematuria, or microscopic hematuria. Rarely, they showed persistent nephropathy with decreased GFR[9]. Finally, 14 children were treated for a mean of 7.5 years with combined therapy consisting of cyclophosphamide, prednisolone, heparin/warfarin, and dipyridamole. The histology abnormalities of the kidney were retrospectively significantly improved[36].

Again the effectiveness of the type of administration (intravenous or oral, alone or an association of drugs) may be questionable. In childhood NS, intravenous cyclophosphamide has shown fewer side effects than oral administration[37]. For this reason, the intravenous cyclophosphamide therapy also could be advised as a good approach for childhood HSP nephritis. Finally, the available literature is not in agreement with the effectiveness of cyclophosphamide. The randomized controlled trial[31] did not show a beneficial effect with time. For the other investigation, we cannot exclude the beneficial effect of the other drugs (dipyridamole and methylprednisolone and prednisolone) used in combination with cyclophosphamide.

## Angiotensin II Inhibitors

Two classes of antihypertensive drugs that block the renin-angiotensin system are in clinical use. They are the angiotensin converting enzyme inhibitors (ACE-Is) and the angiotensin receptor blockers (ARBs). In general, meta-analyses have shown that ACE-Is have the potential to protect against deterioration of renal function in nondiabetic nephropathies[38]. In particular, ACE-Is and ARBs can reduce the urinary protein excretion and ameliorate the GFR in IgA nephropathy[39]. In addition to the antihypertensive effect, angiotensin II inhibition exerts specific effects in the vasculature[40] and the kidney, i.e., decreased intraglomerular pressure and improved glomerular-barrier size selectivity[39].

Unfortunately, very few data are available of the efficacy of ACE-Is in reducing proteinuria in children with HSP nephritis. Only one recent paper highlighted the efficacy of the ACE-I in HSP nephropathy. So for five patients with HSP nephritis, the therapy with ACE-I, combined with fish-oil, was successfully used as a combined therapy, first for hypertension, but also showing a reduction of the protein excretion rate[41]. Therefore, several convincing data are available on the efficacy of angiotensin II inhibitors in protecting kidney function in proteinuric renal disease and are previously reviewed[42]. So the beneficial use of ACE-Is and ARBs might likely be poorly questionable in HSP nephritis.

## Azathioprine or Mycophenolate Mofetil and Steroids

Azathioprine is an immunosuppressant drug. It has been used mostly in association with steroids than alone, in children with crescent HSP nephritis. In a clinical trial, the starting association of azathioprine with intravenous methylprednisolone or with oral prednisone, followed by long-term (9–24 months) alternate-day prednisone, showed a comparable outcome in HSP children[43]. In a retrospective investigation, the results were also confirmed. In particular, azathioprine (2 mg/kg/day, ≈15 months) associated with methylprednisolone (30 mg/kg/day, 3 days) or dexamethasone (5 mg/kg/day), and then followed by oral prednisolone (≈12 months) was able to treat severe HSP nephritis in children[44]. In addition, early treatment showed very important means. In fact, in another retrospective study, azathioprine (1–2 mg/kg/day) and prednisone (1–2 mg/kg/day) were shown to prevent the progression of chronic kidney changes in severe disease[45]. In addition, azathioprine (2 mg/kg/day) for 8 months and steroids, in HSP children (4.4–17 years old) followed for a mean of ≈5 years, may be beneficial in ameliorating histopathological abnormalities and improving the clinical course[46]. Unfortunately, the limit of the investigations performed are that the azathioprine treatment was combined with steroids and only one clinical trial is available. All other studies were designed retrospectively.

Mycophenolate mofetil is an immunosuppressant drug that inhibits the novo purine synthesis and reduces antibody production. This drug is shown as a safe and effective therapy for the treatment of several autoimmune diseases. Therefore, only recently has it been reported as a safe and effective means to treat childhood nephritis. So, in severe forms of IgA nephropathy in children less than 18 years old, mycophenolate mofetil was effective at a dose of 1,200 mg/mq/day[47]. In a clinical trial, the drug was used to treat different nephritis. In this study, the better pediatric dose was 900 mg/mq/day (25 ± 10 mg/kg/day). Promising results were also shown in a single severe case of HSP nephritis[48]. Furthermore, children with prolonged HSP kidney disease, characterized by severe hematuria and proteinuria, were treated with steroids and azathioprine first, and mycophenolate mofetil later. In this retrospective study, the treatment was probably effective[46]. In summary, very few reports are available on mycophenolato mofetil treatment in HSP nephritis. For this reason, the drug would need to be tested more.

### Urokinase and Steroids and Cyclophosphamide

Urokinase performs the digestion of the mesangial matrices of the kidney. For this reason, urokinase should be used with the aim to prevent the mesangial proliferation observed during HSP nephritis in children[49]. The dosages have been discussed by Kawasaki et al.[50,51] in rapidly progressive HSP nephritis. Urokinase was used as pulse therapy, at the dose of 5,000 U/kg/day (maximum 180,000 U) and administrated for 7 consecutive days. Furthermore, in association with methylprednisolone pulse at 30 mg/kg/day (maximum 1 g/day) for 3 consecutive days, it should be effective for patients at risk of nephropathy progression. After 6 months of treatment, a reduction of urinary protein excretion rate and a failure of increased sclerosed glomeruli was observed. Finally, urokinase pulse therapy, in association with pulse steroids, plasmapheresis, or cyclophosphamide, can be suggested as effective in rapidly nephritic disease[50,51]. In summary, these few reports concluded that urokinase, associated with other immunosuppressive drugs, may be an effective measure.

## ADDITIONAL THERAPIES

### Plasmapheresis

Children with rapidly progressive HSP nephritis and nephritic-range proteinuria could be treated with plasmapheresis. Plasmapheresis is a process in which the plasma is removed from blood cells by a cell separator. The blood cells are then returned to the patient, while the plasma, which contains the antibodies, is discarded and replaced with other fluids. In a retrospective study of nine children with rapidly progressive HSP nephritis, the plasmapheresis may have been effective in the improvement of renal function, reduction of proteinuria, and resolution of abdominal pain[52]. In a large multicenter study, 64 children with a severe form of HSP nephritis were followed for 1–23 years. In these children, the early treatment with almost seven sessions of plasma exchanged appeared to delay the progression of the most severe form of nephritis[53]. This technique was also suggested for patients with extensive crescent formation and sudden decline of renal function. The suggested volume exchanged was 50 ml/kg of plasma with 5% human albumin solution for each session, for a total of five treatments of plasmapheresis in 2 weeks. Six children with HSP nephritis, enrolled in an uncontrolled study, received multiple drug therapy combined with plasmapheresis. The combination showed beneficial effect in more rapidly progressive nephritis forms[51]. Unfortunately, there are very few clinical observations in children. Although conclusive results are not available, we cannot underestimate the results of a multicenter study[53]. This investigation showed that the plasmapheresis could be particularly suggested in cases of HSP nephritis with rapidly decline of kidney function.

### Intravenous Immunoglobulin

The intravenous immunoglobulin (IVIG) therapy has been shown to be effective in treating and protecting renal function in adults[54]. This therapy showed a significant decrease in albuminuria as well as in the histological activity index in a follow-up biopsy. In a prospective uncontrolled trial reporting on 13 subjects, the treatment duration was of 9 months with 0.35 ml/kg once a week for 1 month, followed by 0.35 ml/kg every 15 days for a further 8 months. The majority of the patients had a clinical improvement[55]. The possible target of the therapy by immunoglobulin is proinflammatory cytokine. The overproduction of TNF

and IL-6 should be elevated in HSP and reduced after 9 months of immunoglobulin administration[55], however, the efficacy of IVIG in inhibiting the disease progression has been described only in single case reports, as previously reviewed[57]. In conclusion, the beneficial use of IVIG is again questionable and needs to be proven, particularly in children.

## Factor XIII

Considering that the coagulation factors could be altered in sera of HSP children, the substitutive therapy has been also considered. In particular, Factor XIII activity should be decreased in children with a severe form of HSP[58]. In addition, a decreased factor XIII activity along with persistent purpura would increase the risk of developing renal involvement in children older than 7 years with HSP[15]. In a controlled clinical trial, in 24 cases, the intravenous administration of Factor XIII concentrate improved the renal symptoms of the patient after 3 days[59].

## Antioxidants

A chronic oxidative process, triggered by inflammation, contributes to the worsening of renal damage. Both oxidative damage and lipid peroxidation might play an important role also in the pathogenesis of HSP nephritis. In this view, an antioxidant, such as vitamin E, has been considered in the therapy. Unfortunately, a clinical trial did not demonstrate its usefulness. After initiation of lipid peroxidation, the antioxidant vitamin E did not give therapeutic results. In particular, oxidative damage and worsening of clinical course were observed despite significant increases in plasma vitamin E levels[60].

Omega-3 polyunsaturated fatty acids, important active substances of fish oil, are able to attenuate inflammatory responses. Thus, the renoprotective effects of fish oil may be the result of mitigation of glomerular or tubulointerstitial inflammation. Fish oil (1 g, twice daily) and ACE-Is have been tested in five children with HSP nephritis. In particular, the protein excretion rate prior to starting fish oil was 1,041 mg/day and at the last follow-up, the excretion rate was 104 mg/day ($p < 0.05$)[41]. The limitation of this study was that the number of children tested was very low and ACE-Is have a heavy antiproteinuric effect. On the other hand, a recent review clearly showed the very important role of ACE-Is in reducing proteinuria[38].

## CONCLUSIONS

Renal involvement in children with HSP is a frequent finding. Therefore, severe cases of nephritis are less frequently observed. In these severe cases, the institution of early and aggressive immunosuppressive treatment can improve the long-term survival of the kidney.

Therapy of HSP nephritis in children could range from steroids to combined immunosuppressant treatments. The prophylactic treatment for renal complication of patients with HSP has been sometimes suggested, but with conflicting results and ultimately not clearly proven[24]. The treatment of overt HSP nephritis includes steroids and other immunosuppressant drugs. Methylprednisolone pulse therapy and prednisone *per os* are tested drugs. These steroids could be used in combination with other immunosuppressant drugs, such as CyA and cyclophosphamide. Unfortunately, of these two drugs, only cyclophosphamide has demonstrated effectiveness in a recent randomized controlled trial[31]. However, since there are insufficient data and unstructured study designs, ACE-I, azathioprine, mycophenolate mofetil, and urokinase need to be more tested in childhood HSP nephritis. In addition, most studies should be interpreted cautiously as they are drawn from case reports or small series of patients followed for only short periods of time. In addition to drugs, other techniques were used to treat severe forms of nephritis. Of these, plasmapheresis has demonstrated efficacy in a multicenter study in delaying the progression of kidney disease[53]. No convincing studies have been made to date concerning either IVIG, factor XIII administration, antioxidant vitamin E, and fish oil to treat HSP nephritis.

## DECLARATION OF COMPETING INTERESTS

The authors declare that they have no competing interests.
